# The Preparation of a Polyamidoxime–Phosphorylated Cellulose Nanofibrils Composite Aerogel for the Selective Extraction of Uranium from Seawater

**DOI:** 10.3390/nano14151297

**Published:** 2024-08-01

**Authors:** Xiaoying Yang, Mei Cui, Rongxin Su, Renliang Huang

**Affiliations:** 1 Tianjin Key Laboratory for Marine Environmental Research and Service, School of Marine Science and Technology, Tianjin University, Tianjin 300072, China; 2021227002@tju.edu.cn (X.Y.);; 2 State Key Laboratory of Chemical Engineering, Tianjin Key Laboratory of Membrane Science and Desalination Technology, School of Chemical Engineering and Technology, Tianjin University, Tianjin 300072, China; 3 Tianjin Sustainable Novel Materials Co., Ltd., Tianjin 300192, China

**Keywords:** uranium, phosphorylated cellulose nanofibrils, polyamidoxime, adsorption

## Abstract

Uranium is the most important fuel for nuclear power operations, and the safe supply of its resources is the key to the development of nuclear power in China. Because of the complex seawater environment and extremely low uranium concentration, extracting uranium from natural seawater poses a significant challenge. In this study, a polyamidoxime–phosphorylated cellulose nanofibril composite aerogel was prepared as an adsorbent for uranium extraction from seawater. An adsorption kinetics test, equilibrium adsorption isotherm model fitting, an adsorption–desorption cycle test, and a selectivity test were carried out to evaluate the adsorption performance of the composite aerogel for uranium extraction. The adsorption capacities for the initial concentrations of 4 and 8 ppm in uranium-spiked pure water were 96.9 and 204.3 mg-U/g-Ads, respectively. The equilibrium uranium adsorption capacities of uranium-spiked simulated seawater were 38.9 and 51.7 mg-U/g-Ads, respectively. The distribution coefficient *K_D_* of uranium was calculated to be 2.5 × 10^7^ mL/g. The results show that the polyamidoxime–phosphorylated cellulose nanofiber composite aerogels prepared in this study have the advantages of low cost and high uranium selectivity for uranium extraction from seawater.

## 1. Introduction

Uranium is the main fuel used in the nuclear power industry, and its reserves directly threaten the feasibility of nuclear power technology [1,2]. In light of this issue, it is of great significance to find alternate uranium sources for future nuclear power energy supply [1]. The total reserves of uranium in seawater stand at around 4.5 billion tons, which is more than 1000 times that of uranium ore reserves on land [3]. These reserves are expected to fuel nuclear power generation. The separation of uranium from seawater is a complex process because of the high ionic strength of complex metals in the ocean and the low concentration of uranium, limiting the process of this work [3,4]. What is more, seawater is a complex biogeochemical system with a significant amount of competing metal ions, high salinity, and a substantial propensity for marine biofouling, which eventually make the long-term adsorption of uranium from seawater difficult [5,6,7]. Therefore, highly selective uranium adsorption materials are vital to improving the efficiency of uranium extraction from natural seawater.

Uranium exists mainly in the form of uranyl carbonate ([UO_2_(CO_3_)_3_]^4−^) in seawater. The methods used for the separation of uranium from seawater include adsorption, membrane separation, ion exchange, and chemical precipitation [8,9,10]. Adsorption is considered the most effective and economical method for the extraction, desorption, and recycling of uranium in seawater. One of the major aims of this method is to adsorb materials with high selectivity and high capacity. Following years of research and exploration, researchers have prepared a variety of uranium adsorption materials, including organic functional materials, porous carbon materials, metal–organic framework materials, covalent organic framework materials, and mesoporous silica metal oxides [11].

Cellulose nanofibrils are a type of green and renewable material. After Ranby [12] used sulfuric acid to hydrolyze wood and cotton cellulose in 1949 to produce stable cellulose crystals of colloidal size, there has been widespread interest in this type of material. Researchers have successively prepared cellulose nanofibrils, such as microcrystalline cellulose, beet primary cell wall cellulose, encapsulated cellulose, and hyacinth cellulose, from various raw materials using acid hydrolysis. Furthermore, in recent years, cellulose nanofibrils have attracted attention because of their green and environmental protection characteristics [13].

In general, the term cellulose nanofibril signifies that the cellulose extract, or processed material, is nano-sized. Such a material comprises a crystal domain formed from cellulose [14], which is derived primarily from globally ubiquitous green plants and, in part, from animals and bacteria. Compared with cellulose, cellulose nanofibrils have excellent properties, such as high strength, specific surface area, and crystallinity, making them good reinforcing materials when compounded with other materials. Studies have shown that ultrafine fibers can increase the contact area between the adsorbent and seawater during the uranium extraction process [15].

As we know, amidoxime-based materials were first applied by Japanese scholars for uranium extraction from seawater and gradually became the main research direction for this application [16,17,18,19,20,21]. Due to their high adsorption selectivity for uranyl ions, these materials are a suitable nucleophilic ligand for uranium adsorption and the most effective materials for the separation of uranium from the natural seawater [22,23]. However, for amidoxime-based adsorbents, some metal ions (e.g., vanadium, iron, and copper) in seawater compete with uranium-based ions. Vanadium is the most competitive interfering element in the adsorption of uranium by amidoxime groups [24]. Therefore, the adsorption capacity of the adsorbent for uranyl ions is greatly reduced. These factors limit the wider use of amidoxime adsorbents [3,25]. However, amidoxime polymers have been widely used in the past 40 years because of their low cost and ease of handling, and they have been deployed in real seawater extraction tests in the oceans [7]. Some studies have hitherto estimated that the cost to extract 1 kg of uranium from seawater using the best adsorbents lies around USD 150–300. To further reduce the extraction cost, not only should the fabrication cost be kept low, but the adsorbent needs to be mechanically robust to guarantee a long service life.

Some studies have shown that phosphate groups can effectively improve the selectivity of amidoxime groups to uranium because stable chemical bonds can be formed between uranium and phosphate groups [26]. For example, Jackson et al. [27] studied the structure of anhydrous and hydrated complexes of UO_2_^2+^ with phosphate anions H_2_PO_4_^−^, HPO_4_^2−^, and PO_4_^3−^ using DFT [28] and MP2 [29] molecular orbital theories. They found that these ions undergo monodentate and bidentate binding with uranyl ions, which indicates that phosphoric acid grafting can improve the adsorption selectivity of uranyl ions [30,31,32]. As phosphorylated cellulose nanofibers (PCNFs) are prepared during the early stages of production, there are numerous preparation methods available, such as a simple and green production process [33], which yields not only excellent properties, such as a high specific surface area and strength, unique to cellulose nanofibers but also high uranium selective adsorption performance due to the existence of phosphate radicals.

The purpose of this study was to combine PCNFs and polyamidoxime (PAO) to synthesize composite aerogels at a low cost and with high uranium selectivity, optimize the aerogel composition and preparation process parameters, and evaluate the mechanical strength and uranium adsorption and separation performance of aerogels.

## 2. Materials and Methods

### 2.1. Materials

Never-dried softwood bleached kraft pulp from Pinus radiata (Arauco; Yangrun Trading Co., Ltd., Dalian, China) was used. Pure water (Conductivity: 27.3 μs/cm) was used for all the preparations. All reagents involved in this study are listed in the table below (Table 1). All chemicals were used without any further purification. Among them, Analytical Reagent is represented by AR, Guaranteed Reagent is represented by GR, and molecular weight is represented by *M_w_.*

### 2.2. Synthesis of Composite Materials

#### 2.2.1. Preparation of PCNFs

**Figure 1:**
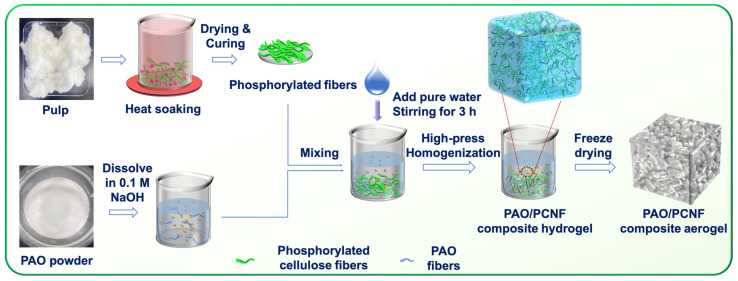
Schematic of the preparation of the PAO-PCNF composite aerogel.

The PCNFs were prepared using hot-dip pretreatment according to Chen [33]. As shown in Figure 1, raw cellulose fibers (50 g) were crushed into paper scraps with a pulverizer and soaked completely in a beaker containing 21.3 g of NH_4_H_2_PO_4_ and 59.32 g of urea aqueous solution with a solid content of 22 wt%. The paper scraps were then soaked at 80 °C for 1 h, removed from the beaker, placed in a sieve, and dried in an electric thermostatic drying oven (WGLL-65BE; Tianjin Taisite Instrument Co., Ltd., Tianjin, China) at 105 °C for 4 h. The dried sample was then removed, the temperature of the electric thermostatic drying oven was set to 150 °C, and then, the sample was placed in the electric thermostatic drying oven again for 20 min to allow for solidification. Afterward, it was immediately removed from the oven and placed in the fume hood to fully volatilize the ammonia generated by the reaction. The solidified sample was placed in a beaker, pure water was added, and the sample was dispersed again. A stirring paddle was used to stir the sample for 30 min. A vacuum suction filter device was installed, and the suction filter operation was carried out with a 500-mesh nylon cloth until the conductivity of the filtrate was less than 50 μS∙cm^−1^ and the washing procedure was complete. Pure water was re-added to achieve 1 kg total weight, the pH was adjusted to 9.5 with 30 wt% sodium hydroxide solution, and the sample was beaten with a beater and then stirred in a hydrogel state for 1 h. The solid content was determined to prepare the composite hydrogel with poly(amideoxime) and calculate the PCNF dosage.

#### 2.2.2. Synthesis of PAO

The procedure utilized for the synthesis of poly(amideoxime) using polyacrylonitrile was as outlined by Liu [11]. NH_2_OH∙HCL (5.5 g) was dissolved in a DMF (60 mL) solvent in a round-bottom flask and then heated to 45 °C using a water bath with magnetic stirring until complete dissolution was achieved. Sodium bicarbonate (3.8 g) and sodium hydroxide (1 g) were then added to the mixed solution and stirred for at least 180 min. PAN (4.2 g) was added to the solution and dissolved completely. The mixed solution was then heated to 65 °C for 24 h. Next, sodium bicarbonate (1.9 g) and sodium hydroxide (0.5 g) were added, and the reaction continued at 65 °C for 12 h. The mixture was cooled to 25 °C and then centrifuged (10,000 rpm for 20 min). The collected PAO supernatant was then dispersed in pure water to precipitate a white floc, which was subsequently dried at 60 °C for 12 h in a vacuum drying oven (DZF-6050; Tianjin Dongsheng Keyi Co., Ltd., Tianjin, China). Lastly, the prepared PAO powders were collected after grinding.

#### 2.2.3. Preparation of Aerogel Adsorbents

For the preparation of the PAO-PCNF composite aerogels, four proportions of composite aerogels were created by mixing 1, 2, 4, and 20 g of PAO powder with 20 g of PCNFs, before which the PAO powder was dissolved in 50, 100, 200, and 700 mL of 0.1 M of NaOH solution, respectively, and then by mixing them with pure water to a suspension of 1.5 kg total mass (Table 2). The suspension was mixed with a stirring paddle for 3 h and homogenized with a homogenizer 6–8 times under high pressure (600–1000 bar). Next, the solid content was determined and adjusted to 1 wt% with pure water. The result was four hydrogel samples with different composite ratios of PCNFs of PAO: 1:1, 5:1, 10:1, and 20:1.

For the PCNFs aerogels, phosphorylated cellulose in a similar hydrogel state was directly homogenized under high pressure 6–8 times; afterward, the PCNF hydrogel was used to determine the solid content and adjusted to 1 wt% with pure water. The four composite and one PCNF hydrogel samples were freeze dried, and the corresponding aerogel samples were obtained.

### 2.3. Characterization

A field emission scanning electron microscope (FE-SEM; S-4800; Hitachi, Japan) equipped with an energy-dispersive spectrometer (EDS) was used to characterize the morphology and elemental spectra of the samples. The mechanical strength of each sample was tested using an electronic universal testing machine (CMT6103; MTS, Shanghai, China). The width and shape of the PCNFs were studied via transmission electron microscopy (TEM; JEOL, JEM-1400Flash, Tokyo, Japan) at a 120 kV acceleration voltage. Before observation, the PCNFs were subjected to phosphotungstic acid negative staining.

The transmittance of 0.1 wt% PCNF dispersion was measured using a UV−vis spectrophotometer (TU1810; Beijing Boxi General Instrument Co., Ltd., China) as an indirect index of the nanofibrillation yield (with the wavelength range of 300–800 nm). The transmittance measurement of the calibration sample with pure water was used as a blank. A UV−vis spectrophotometer (TU1810; Beijing Boxi General Instrument Co., Ltd., Beijing, China) was used to determine the optical adsorption spectrum and measure the absorbance of the solution at the maximum adsorption peak of 651 nm to determine the uranium concentration in the solution. The detection limit for uranium ions in samples is ~0.5 ppm.

The charge density of the PCNFs was examined via conductometric titration according to the Canadian Standard CSA Z5100-17 [34]. The PCNFs were dispersed in pure water at a concentration of 0.1 wt %, and the conductivity was adjusted to 700 μS·cm^−1^ using 0.1 M HCl. The charge density was then measured via titration with 0.1 M NaOH. Each sample was titrated three times. The zeta potential (0.1 wt%) of the PCNF dispersions was measured using a nanometer-size potentiometer (Zetasizer Lab; Malvern Instruments, Malvern, UK). The verification of the successful synthesis of PAO was carried out through Fourier transform infrared spectroscopy (FTIR; Nicolet iS20; Thermo Fisher Scientific, Waltham, MA, USA) under transmission mode.

Unless otherwise stated, the adsorption capacity of uranium in simulated seawater with uranium added was analyzed using the Arsenazo III assay [35]. Competitive adsorption experiments were carried out in simulated seawater with a competitive ion concentration of 100× natural seawater; the concentration of the adsorbed uranium and other interfering metal ions was measured using inductively coupled plasma optical emission spectroscopy (ICP-OES; 5110; Agilent, Santa Clara, CA, USA). The porosity, pore size distribution, pore volume, pore area, and other data on the adsorbents were measured using a mercury porosimeter (AutoPore V 9620; Micromeritics, Norcross, GA, USA). The elemental composition of the adsorbent surface before and after adsorption was measured using X-ray photoelectron spectroscopy (XPS; Nexsa G2; Thermo Fisher Scientific, Waltham, MA, USA).

### 2.4. Determination of Uranium Adsorption

#### 2.4.1. Preparation of Uranium-Spiked Simulated Seawater

Uranium-spiked simulated seawater was produced by dissolving uranium hexahydrate nitrate [UO_2_(NO_3_)_2_·6H_2_O; AR] in simulated seawater (pH = 8) consisting of 25.6 g/L NaCl and 0.198 g/L NaHCO_3_. In each simulated adsorption experiment, 10 mg of adsorbent was immersed in 0.5 L of uranium-spiked simulated seawater. The adsorption process was performed in the water bath shaker unless otherwise indicated.

#### 2.4.2. Adsorption Kinetics of Uranium in Uranium-Spiked Pure Water

Uranium-spiked pure water with different initial uranium concentrations (4 and 8 ppm, pH = 8) was prepared for the adsorption kinetics study. Aliquots were removed from the solution at appropriate adsorption time intervals and analyzed using the Arsenazo III assay. The uranium adsorption capacity was calculated using the following equation (Equation (1)):

(1) $$Q_{t}=(C_{0}-C_{t})\times \frac{V}{m}$$

where *Q_t_* is the uranium adsorption amount at exposure time *t* (mg-U/g-Ads); *C*_0_ and *C_t_* are the uranium concentrations in the simulated seawater at exposure time 0 and *t*, respectively, (ppm); *V* is the volume of the simulated seawater (L); and *m* is the adsorbent mass (g). When the equilibrium state is reached, *Q_t_* and *C_t_* are substituted by *Q_e_* and *C_e_*, respectively.

The pseudo-second-order (Equation (2)) and pseudo-first-order (Equation (3)) kinetic models were applied to analyze the uranium adsorption kinetic data [36].

(2) $$\frac{t}{Q_{t}}=\frac{1}{k_{2}Q_{e}^{2}}+\frac{t}{Q_{e}}$$

Here, *k*_2_ is the rate constant (g mg^−1^ min^−1^).

(3) $$ln(Q_{e}-Q_{t})=lnQ_{e}-k_{1}t$$

Here, *k*_1_ is the rate constant (min^−1^).

#### 2.4.3. Equilibrium Adsorption Isotherm Test

During this stage, 0.5 L samples of uranium-spiked simulated seawater with different initial concentrations of uranium (2, 4, 8, 16, and 32 ppm, pH = 8) were prepared for equilibrium adsorption isotherm experiments. After the solution was stirred at room temperature for 24 h, aliquots were removed and analyzed using the Arsenazo III assay. The uranium adsorption capacity was calculated using Equation (1). The equilibrium isotherm data were fitted using the Langmuir (Equation (4)) and Freundlich (Equation (5)) models [37].

(4) $$\frac{C_{e}}{Q_{e}}=\frac{C_{e}}{Q_{m}}+\frac{1}{k_{3}Q_{m}}$$

Here, *C_e_* is the equilibrium concentration (ppm), *Q_m_* is the saturated adsorption amount (mg/g), and *k*_3_ is an equilibrium constant related to the binding strength (L/mg).

(5) $$lgQ_{e}=lgk_{4}+\frac{1}{n}lgC_{e}$$

Here, the constant *k*_4_ is an approximate indicator of the adsorption capacity (mg/g/(ppm)^n^), and 1/*n* is a function of the strength of adsorption in the adsorption process.

#### 2.4.4. Selective Adsorption from Uranium-Spiked Simulated Seawater at 100× Natural Seawater

During this stage, we added the co-existing metal ions (UO_2_^2+^, VO_3_^−^, Co^2+^, and Ni^2+^) to the simulated seawater. The concentration of each interfering element was 100 times that of real seawater. The specific concentrations are 0.33 ppm UO_2_^2+^, 0.2 ppm VO_3_^−^, 0.002 ppm Co^2+^, and 0.5 ppm Ni^2+^. After stirring the solution at 25 °C for 24 h, we removed aliquots from the mixture and analyzed them using ICP-OES. The distribution coefficient *K_D_* (mL/g) of the adsorbents to metal elements was calculated using Equation (6):

(6) $$K_{D}=[(C_{0}-C_{e})\times V]/(C_{e}\times m)\times 10^{6}$$

where *C*_0_ is the uranium concentration in the simulated seawater at exposure time 0 (ppm), *C_e_* is the equilibrium concentration (ppm), *V* is the volume of the simulated seawater (L), and *m* is the adsorbent mass (g).

#### 2.4.5. Desorption Test

The uranium-spiked samples were immersed in an eluent composed of 200 mL 1 M Na_2_CO_3_ and 0.1 M H_2_O_2_ for the regeneration of adsorbents. Then, aliquots of the eluent were collected and analyzed using the Arsenazo III assay to determine the uranium concentration.

## 3. Results and Discussion

### 3.1. Preparation and Characterization of Adsorbents

#### 3.1.1. Synthesis and Characterization of PAO

**Figure 2:**
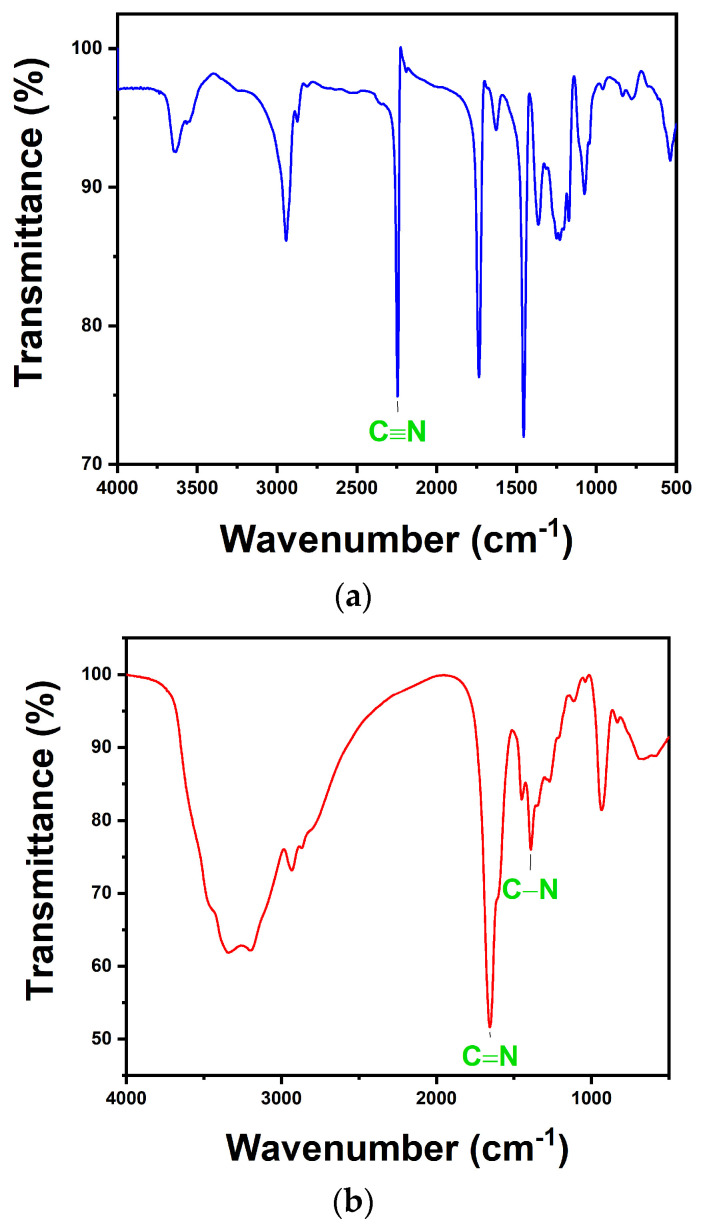
FTIR spectra of (**a**) raw PAN and (**b**) product PAO.

The successful synthesis of PAO was verified by the appearance of characteristic peaks during FTIR analysis. The infrared spectrum of PAN powder shown in Figure 2a depicts the characteristic peak of (C≡N) at 2245 cm^−1^. The infrared spectra of the synthesized PAO powder shown in Figure 2b depict characteristic peaks corresponding to (C=N), (C–N), and (N–O) at 1657, 1391, and 935 cm^−1^, respectively. It can be seen that the characteristic peak of (C≡N) in PAN disappears, and the appearances of (C=N), (C–N), and (N–O) prove that PAN has been successfully transformed into PAO.

#### 3.1.2. Preparation and Characterization of PCNFs

**Figure 3:**
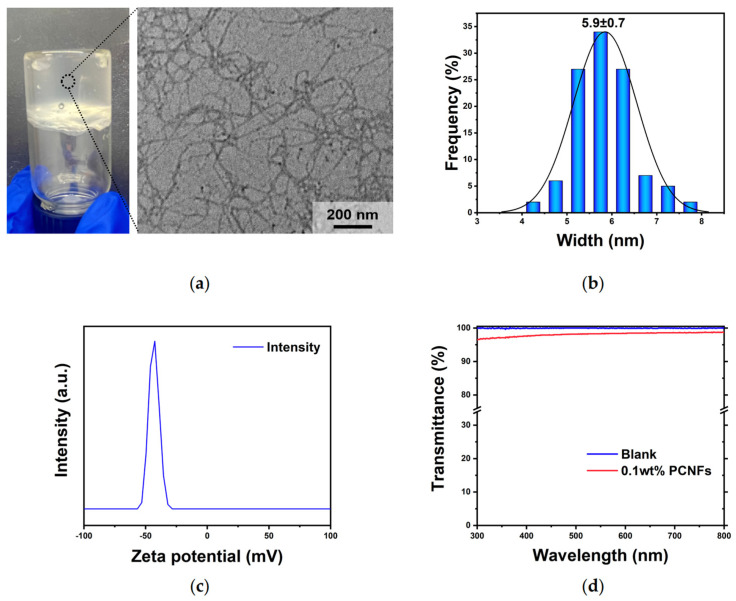
Characterization of the PCNFs. (**a**) Photograph of hydrogels (0.5 wt %) and TEM image of the acquired 0.001 wt% PCNFs at a magnification of 25k×; (**b**) width distributions; (**c**) zeta potential distribution; (**d**) light transmittance.

The TEM image of the 0.001 wt% PCNF hydrogel shown in Figure 3a and the width distribution (Figure 3b) analyzed using Image J software (Version 1.8.0) indicated that the individualized PCNFs exhibited a relative cross-section with average widths of 5.5−6.5 nm. The charge density of the 0.25% PCNFs measured via conductometric titration was 3.07 mmol∙g^−1^.

The stability of the PCNF dispersion generally results from electrostatic repulsion because of the charged groups at the surfaces. The zeta potential of the PCNF dispersion, displayed in Figure 3c, peaked at −43.1 mV, which confirms its colloidal stability.

Transmittance, which depends on the particular wavelength, is related to the width of the nanofiber because light scatters more when the wavelength is close to the particle diameter. The PCNF dispersion (0.1 wt%) shown in Figure 3d exhibits high UV–vis transmittance (>96%) in the 300–800 nm wavelength range. The transmittance of PCNFs prepared at a wavelength of 550 nm was found to be 98.3%, which further indicates that the dispersion effect of PCNFs is considerable.

#### 3.1.3. Characterization of PAO-PCNF Composite Aerogels

**Figure 4:**
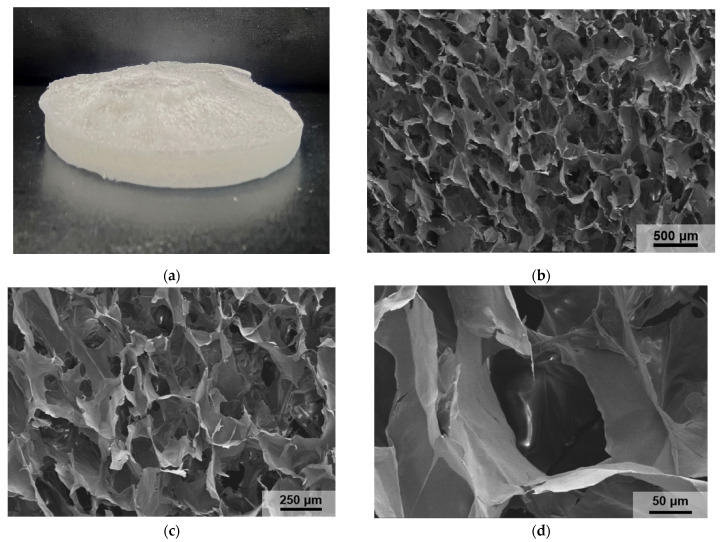
(**a**) Photograph of the PAO-PCNF aerogel and SEM images of a section acquired at magnifications of (**b**) 30k×, (**c**) 60k×, and (**d**) 350k×.

**Figure 5:**
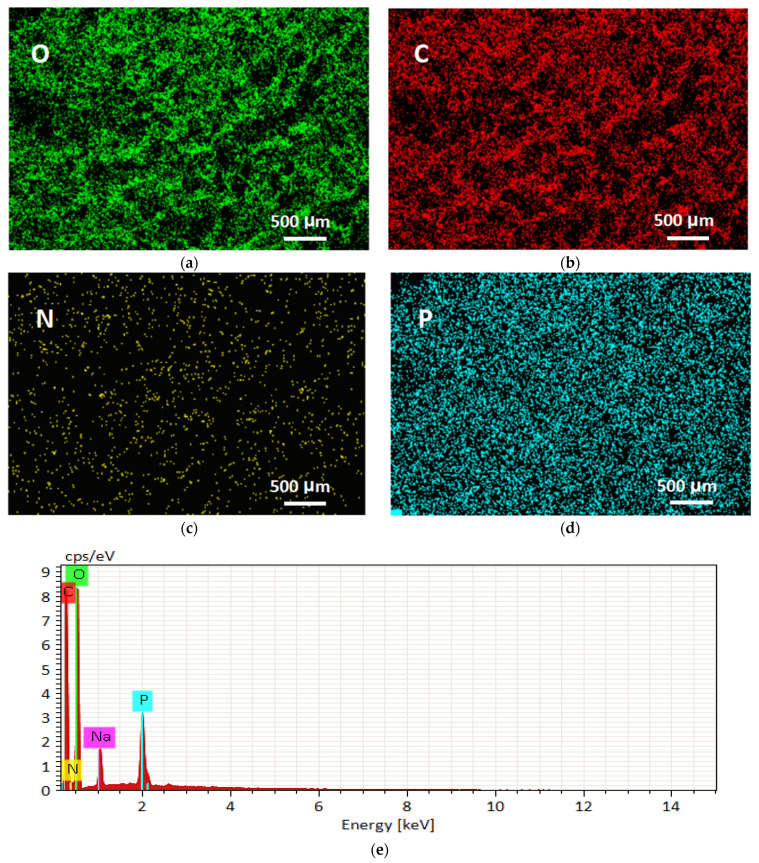
EDS images of the PAO-PCNF aerogels at a magnification of 30k× showing the distribution of (**a**) O, (**b**) C, (**c**) N, and (**d**) P; (**e**) the content map of each element.

The SEM images of the PAO-PCNF composite aerogels in Figure 4a,b (the ratio of composite aerogels is PAO/PCNF = 1:5 unless otherwise specified below) show the microscopic morphology and structure of the PAO-PCNFs composite aerogels, revealing a large number of irregular pore structures with pore diameters of 100–200 μm. The picture in Figure 4c shows that the composite aerogel is a white, uniform, and very light sponge-like mass. The EDS images in Figure 5a–d show a large number of O, C, N, and P elements distributed in the aerogels, with a fairly even scattering on the aerogel surface. Figure 5a–d and the content map of each element shown in Figure 5e indicate that the CNFs were successfully phosphorylated. Table 3 shows the distribution of elements in the mapping graph.

**Figure 6:**
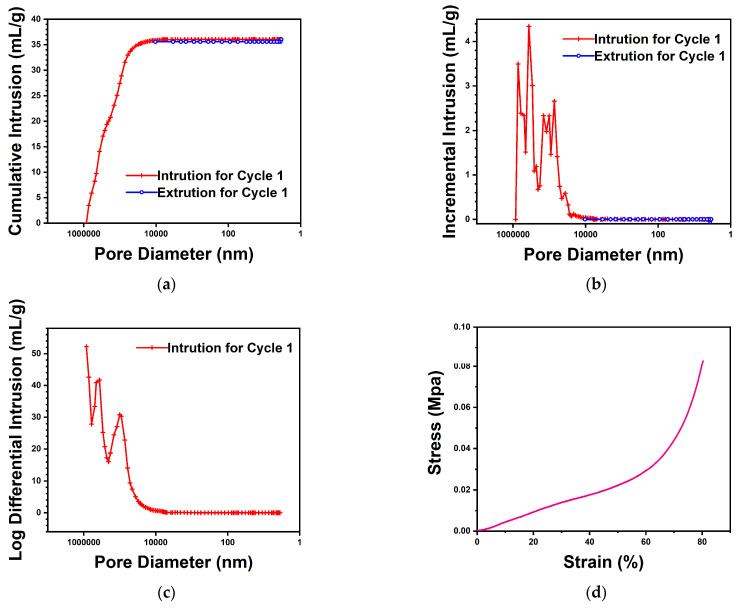
Mercury intrusion porosimetry test results. (**a**) Cumulative intrusion vs. pore size; (**b**) incremental intrusion vs. pore size; (**c**) log differential intrusion vs. pore size; (**d**) stress–strain diagram.

In Figure 6a–c, the mercury porosimeter test results show that the total intrusion volume is 36.0 mL/g; the total pore area is 1.2 m^2^/g; the average pore diameter (4V/A) is 123 μm; and the porosity is 89.5%.

Figure 6d presents the stress–strain curve plotted from the results of the compression tests. The original area of sample A was 40 mm^2^, the compressive modulus *E_c_* was 0.03 Mpa, and the compressive strength *σM* was 0.08 Mpa.

### 3.2. The Absorbance of Uranium-Spiked Seawater

**Figure 7:**
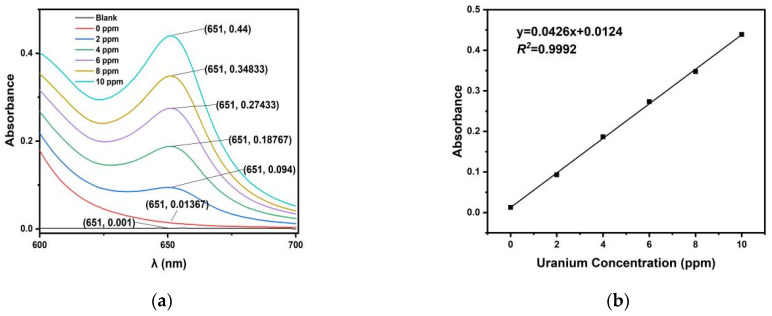
The absorbance of uranium-spiked seawater. (**a**) Absorbance vs. λ in uranium-spiked ultrapure water and (**b**) the curvilinear regression of absorbance–uranium concentration in uranium-spiked simulated seawater.

The prepared 1000 ppm uranium solutions of 0, 20, 40, 60, 80, and 100 μL were each poured into a 10 mL volumetric flask, and the volumes were fixed with simulated seawater to 10 mL, producing 0, 2, 4, 6, 8, and 10 ppm uranium-spiked simulated seawater. The chromogenic reagent prepared according to the Arsenazo III assay was scanned using a UV–vis spectrophotometer in the wavelength range of 600–700 nm. The absorbance curve is shown in Figure 7a. The wavelength at 651 nm is the adsorption peak of uranium. Figure 7b shows the absorbance at 651 nm as a function of the uranium concentration. The test methods used for the absorbance of uranium-spiked pure water and the eluent were as previously stated.

### 3.3. The Effect of Composite Ratio on Uranium Adsorption

**Figure 8:**
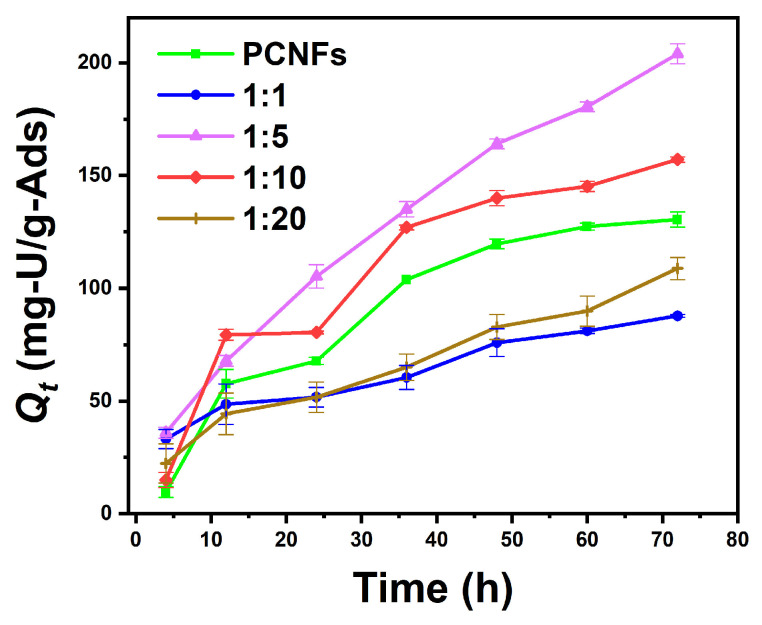
Changes in the uranium adsorption capacity of aerogels in uranium-spiked pure water with different composite ratios over time (mass of adsorbent is 10 mg; volume of uranium solution is 0.5 L).

Figure 8 shows that the uranium adsorption capacity of the composite aerogel with a PCNF/PAO ratio of 5:1 can reach 204.3 mg-U/g-Ads in uranium-spiked pure water for 72 h; when the PCNF/PAO ratio increases to 10:1, the adsorptive capacity of uranium increases because the amide oxime group has a high adsorptive capacity for uranium in PAO. The composite aerogel with a PCNF/PAO ratio of 1:1 exhibited the worst uranium adsorption performance, which may be due to the significant dissolution of PAO. The adsorption of uranium by the PCNFs was better than that by the composite aerogel with a PCNF/PAO ratio of 20:1. We speculate that the phosphate group in the PCNFs provides more uranium binding sites than a small number of amidoxime groups because of the extremely low PAO content. Table 4 summarizes a comparison of the adsorption capacities of various adsorbents in the relevant literature; the maximum uranium adsorption capacity of the PAO-PCNF composite aerogel was close to or higher than the values in the previous studies.

### 3.4. Uranium Adsorption Kinetics in Uranium-Spiked Pure Water

**Figure 9:**
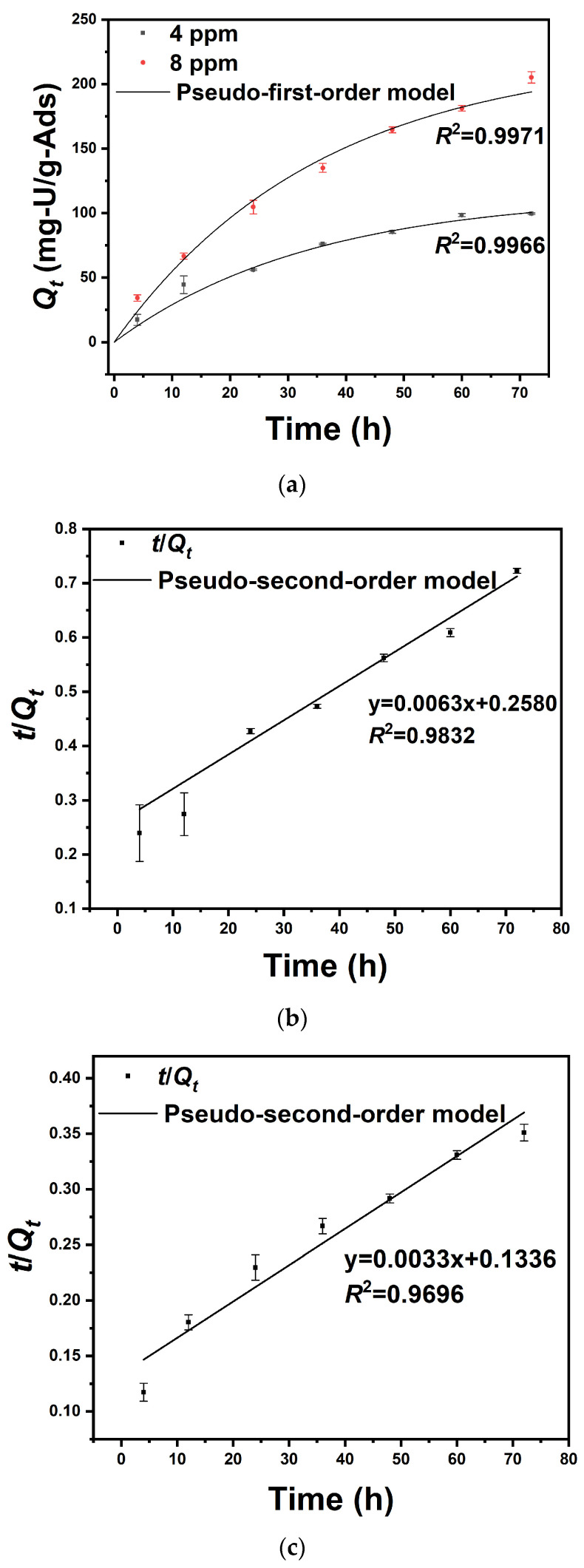
Adsorption kinetics data and corresponding fitting curves based on (**a**) the pseudo-first-order model in 0.5 L uranium-spiked pure water with different initial uranium concentrations (4 and 8 ppm, 10 mg adsorbent), (**b**) the pseudo-second-order model in 0.5 L uranium-spiked pure water with a 4 ppm initial uranium concentration (10 mg adsorbent), and (**c**) the pseudo-second- and -first-order models in uranium-spiked pure water with an 8 ppm initial uranium concentration (10 mg adsorbent).

As shown in Figure 9a, 0.5 L of the uranium-spiked pure water with initial concentrations of 4 and 8 ppm of uranium reached adsorption equilibrium after 72 h. The adsorption capacities were measured as 96.91 ± 0. 61 and 204.3 ± 4. 37 mg-U/g-Ads, respectively. Figure 9b,c show that the regression coefficient of pseudo-second-order kinetic fitting is smaller than that of pseudo-first-order kinetic fitting, indicating that the adsorption of the adsorbent prepared in this study is more in line with the pseudo-first-order adsorption kinetic model and, thus, the adsorption rate is controlled by diffusion steps. In other words, the adsorption reaction rate is proportional to the first order of adsorbent concentration. As shown in Table 5, the theoretical value of saturated adsorption obtained by fitting the pseudo-first-order kinetic model is 114.4 mg/g and 223.2 mg/g, which is similar to the experimental results.

### 3.5. Equilibrium Adsorption Isotherm Test in Uranium-Spiked Simulated Seawater

**Figure 10:**
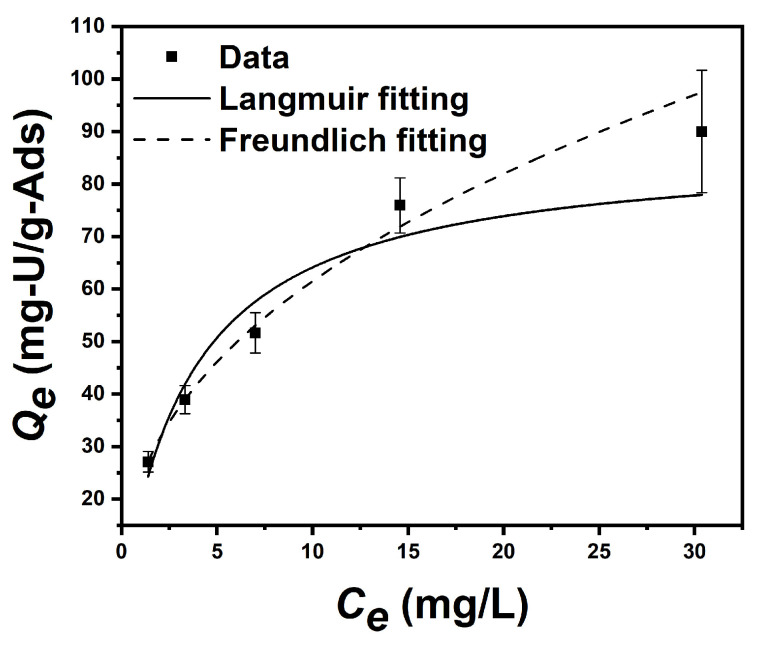
Equilibrium isotherm adsorption data of adsorbents and the corresponding fitting curves based on the Langmuir and Freundlich models, respectively (mass of adsorbent is 10 mg, volume of uranium solution is 0.5 L).

As shown in Figure 10, the equilibrium uranium adsorption capacities of uranium-spiked simulated seawater for the initial concentration of 2 ppm, 4 ppm, 8 ppm, 16 ppm, and 32 ppm were 27.1 ± 2.0, 38.9 ± 2.7, 51.7 ± 3.9, 75.9 ± 5.3 mg, and 90.0 ± 11.7 mg-U/g-Ads, respectively (the initial pH is 8, and the equilibrium pH is 8.5; T = 298.15 K). After calculation, the parameters of the Langmuir model and the Freundlich model can be obtained, shown as Table 6. This adsorption behavior is more consistent with the Freundlich model than with the Langmuir model, which indicates that the adsorption process was not dominated by monolayer chemical adsorption.

### 3.6. Desorption Test in Uranium-Spiked Simulated Seawater

**Figure 11:**
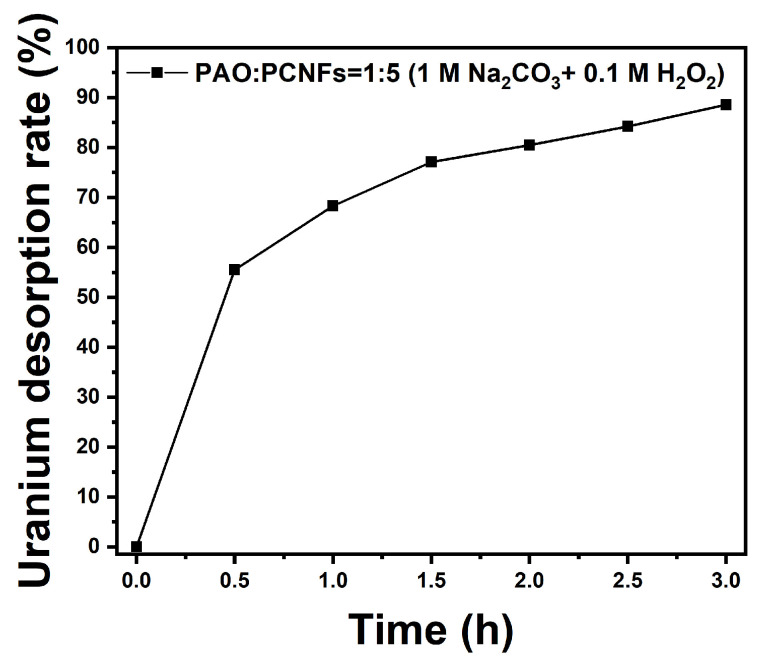
Uranium desorption rate of adsorbents in elution composed of Na_2_CO_3_ (1 M) and H_2_O_2_ (0.1 M).

As shown in Figure 11, nearly half of the uranium was eluted into the eluent in the first half-hour of the experiment, and thereafter, the elution rate slowed down. After desorption for 3 h, the elution rate reached 88.6%, indicating that the uranium bound by the adsorbent was easy to elute. The above results show that the adsorbent can be reused.

### 3.7. Selective Adsorption from Uranium-Spiked Simulated Seawater at 100× Natural Seawater

**Figure 12:**
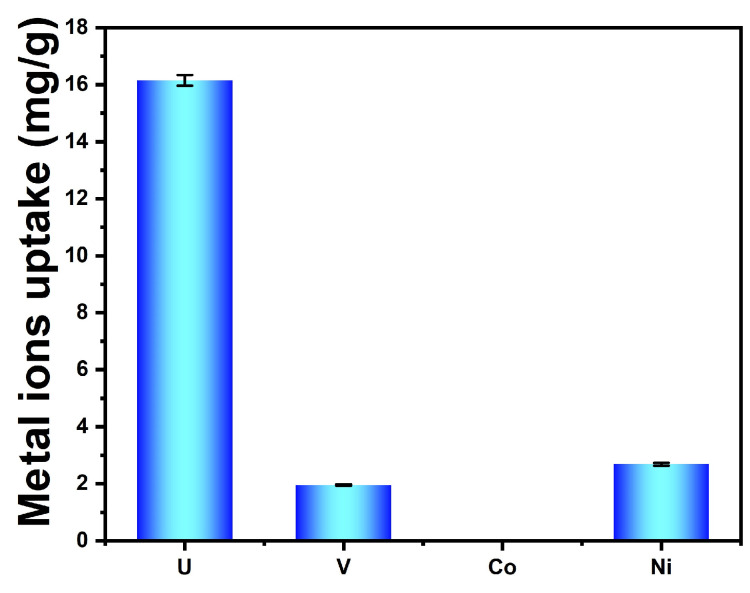
Selective adsorption performance of adsorbent for uranium and interfering metal ions at 100× their natural concentration in seawater.

As shown in Figure 12, the adsorbent produced in this study exhibited extremely high selectivity for uranium adsorption and did not adsorb interfering ions of Co^2+^. The adsorption capacity of the interfering ion Ni^2+^ was less than half of UO_2_^2+^. The adsorption capacity of the interfering ion VO_3_^−^, which is the most competitive with uranium in the extraction of uranium from seawater, was almost less than one-third that of UO_2_^2+^. According to Equation (6), the distribution coefficient *K_D_* of uranium can be calculated as 2.5 × 10^7^ mL/g. The distribution coefficients *K_D_* of vanadium and nickel ions can also be calculated as 6.1 × 10^6^ mL/g and 6.0 × 10^6^ mL/g.

### 3.8. The Mechanism of Uranium Adsorption

For the composite aerogel prepared in this work, it has a large number of pore structures, which provide the physical conditions for the adsorbent to be in full contact with the groups with high affinity for uranyl ions during the adsorption process. On the one hand, the phosphate groups in PCNFs have an affinity for UO_2_^2+^. The adsorption by PCNFs is attributed to the strong electrostatic interactions between the oppositely charged phosphate groups and the UO_2_^2+^ ion in solution [41]. The P=O and P-O bonds of phosphate can effective coordinate with the U (VI) ion to form a stable chelate complex [30]. On the other hand, the adsorption of U(VI) by the PAO-PCNF composite aerogel through the complexation of U(VI) with the imine and hydroxyl in the AO group (−C(NH_2_)=NOH) of PAO implies that the PAO binds to uranium(VI) through the η^2^-N,O mode [42].

## 4. Conclusions

In this study, PAO-PCNF composite aerogels were produced and applied for uranium extraction from seawater. By investigating the influence of the ratio of PCNFs to PAO on the uranium adsorption capacity and comparing the results with pure PCNF aerogels, we found that the composite aerogel with a PCNF/PAO ratio of 5:1 showed the best uranium adsorption performance. Therefore, the adsorption tests were carried out using this ratio.

The uranium adsorption kinetics of this adsorbent in uranium-spiked pure water are more in line with the pseudo-first-order kinetic model than with the second-order model. The initial concentrations of 4 and 8 ppm uranium-spiked pure water reached adsorption equilibrium after 72 h, with adsorption capacities of 96.9 ± 0.6 and 204.3 ± 4.4 mg-U/g-Ads, respectively.

The results of equilibrium adsorption isotherm fitting in uranium-spiked simulated seawater show that the adsorption process is more consistent with the Freundlich model than with the Langmuir model, indicating that the adsorption process does not constitute monolayer adsorption. The equilibrium uranium adsorption capacities of uranium-spiked simulated seawater at initial uranium concentrations of 2, 4, 8, 16, and 32 ppm correspond to 27.1 ± 2.0, 39.0 ± 2.7, 51.7 ± 3.9, 75.9 ± 5.3, and 90.0 ± 11.7 mg-U/g-Ads, respectively.

The test results of the uranium desorption cycle in uranium-spiked simulated seawater show that after 3 h of desorption, the adsorbed uranium was almost completely released into the eluent, indicating that the adsorbent could be reused.

The results of the uranium selectivity test in uranium-spiked simulated seawater show that the adsorbent has excellent selectivity for uranium. The distribution coefficient *K_D_* of uranium can be calculated as 2.5 × 10^7^ mL/g.

In summary, this adsorbent produced from low-cost pulp, PAN, and other raw materials demonstrated proficient selective adsorption of uranium. It has application value in uranium extraction from seawater because of its low cost and high selectivity.

## Figures and Tables

**Table 1:** Reagent, specification, and manufacturer thereof.

Reagent	Specification	Manufacturer
Polyacrylonitrile (PAN)	*M_w_* = 150,000, 99%, AR	Macklin
Hydroxylamine hydrochloride (NH_2_OH·HCl)	99%, AR	Macklin
N,N-Dimethylformamide (DMF)	≥99.8%, ACS	Macklin
Arsenazo III	95%, AR	Macklin
Sodium hydroxide (NaOH)	99.5%, AR	Kermel
Sodium chloride (NaCl)	≥99.5%, AR	Macklin
Sodium bicarbonate (NaHCO_3_)	≥99.8%, AR	Aladdin
Uranium hexahydrate nitrate (UO_2_(NO_3_)_2_·6H_2_O)	AR	Macklin
Ammonium phosphate monobasic (NH_4_H_2_PO_4_)	99%, AR	Meryer
Urea (99%)	AR	Meryer
Hydrogen peroxide (H_2_O_2_)	30%, GR	DAMAO
Anhydrous sodium carbonate (Na_2_CO_3_)	≥99.8%, GR	Feng Chuan
Hydrochloric acid (HCl)	0.1 M	Tianjin Jinke Fine Chemical Research Institute
Nickel chloride titration solution (NiCl_2_)	0.5 M	Aladdin
Sodium metavanadate (NaVO_3_)	99.0%, AR	Macklin
Cobalt chloride (CoCl_2_∙6H_2_O)	99.0%, AR	Feng Chuan
Concentrated hydrochloric acid (HCl)	AR	Feng Chuan

**Table 2:** The amounts of raw materials for four proportions of composite aerogels.

PAO/PCNFs	Mass of PAO (mg)	Mass of PCNFs (mg)	Volume of 0.1 M NaOH (mL)	Total Mass (kg)
1:1	20	20	700	1.5
1:5	4	20	200	1.5
1:10	2	20	100	1.5
1:20	1	20	50	1.5

**Table 3:** Parameters of the breakthrough curves.

Element	Atomicity	Normalized Mass (%)	Atom (%)
O	8	47.03	42.68
C	6	39.45	47.70
N	7	4.75	4.93
Na	11	3.58	2.26
P	15	5.18	2.43
Total		100.00	100.00

**Table 4:** Comparison of the adsorption capacities of various adsorbents for uranium.

Adsorbent	Initial Uranium Concentration (ppm)	Volume of Solution (L)	Adsorbent Dose (g)	pH	*Q_e_* (mg/g)	References
Kelp-like electrospun nanofibers immobilized with bayberry tannin	80	0.05	0.02	5.5	170.1	[38]
Bioinspired hierarchical porous membrane	8	0.2	0.002	5.5	124.2	[39]
Phosphate-functionalized polyethylene	50	1	0.2	8.2	173.8	[40]
Chitosan crosslinked PAO aerogel	100	0.025	0.01	6	203.4	[41]
PAO-PCNF composite aerogel	8	0.5	0.01	7	204.3	This work

**Table 5:** Kinetic parameters of pseudo-first-order and pseudo-second-order kinetic models of PAO-PCNF composite aerogel.

Pseudo-First-Order Kinetic	Initial Uranium Concentration (ppm)	*K*_1_ (min^−1^)	*Q_e_* (mg/g)	*R* ^2^
	4	2.9 × 10^−2^	114.4	0.9966
	8	2.8 × 10^−2^	223.2	0.9971
Pseudo-second-order kinetic	Initial uranium concentration (ppm)	*K*_2_ (g mg^−1^ min^−1^)	*Q_e_* (mg/g)	*R* ^2^
	4	1.5 × 10^−4^	158.5	0.9832
	8	8.0 × 10^−5^	305.8	0.9696

**Table 6:** Parameters of Langmuir isothermal adsorption model and Freundlich isothermal adsorption of PAO-PCNF composite aerogel.

Langmuir	*k*_3_ (L/mg)	*Q_m_* (mg/g)	*R* ^2^
	0.278	87.14	0.9305
Freundlich	*k*_4_ (mg/g/(ppm)^n^)	*1/n*	*R* ^2^
	23.681	0.414	0.9902

## Data Availability

The data presented in the following study are available from the first author upon request.
